# A New Class of Small Molecule Inhibitor of BMP Signaling

**DOI:** 10.1371/journal.pone.0062721

**Published:** 2013-04-30

**Authors:** Caroline E. Sanvitale, Georgina Kerr, Apirat Chaikuad, Marie-Christine Ramel, Agustin H. Mohedas, Sabine Reichert, You Wang, James T. Triffitt, Gregory D. Cuny, Paul B. Yu, Caroline S. Hill, Alex N. Bullock

**Affiliations:** 1 Structural Genomics Consortium, University of Oxford, Oxford, United Kingdom; 2 Laboratory of Developmental Signalling, Cancer Research UK London Research Institute, London, United Kingdom; 3 Department of Medicine Cardiovascular Division, Brigham and Women’s Hospital and Harvard Medical School, Boston, Massachusetts, United States of America; 4 Laboratory for Drug Discovery in Neurodegeneration, Brigham and Women’s Hospital and Harvard Medical School, Cambridge, Massachusetts, United States of America; 5 Botnar Research Centre, University of Oxford, Oxford, United Kingdom; SUNY Upstate Medical University, United States of America

## Abstract

Growth factor signaling pathways are tightly regulated by phosphorylation and include many important kinase targets of interest for drug discovery. Small molecule inhibitors of the bone morphogenetic protein (BMP) receptor kinase ALK2 (ACVR1) are needed urgently to treat the progressively debilitating musculoskeletal disease fibrodysplasia ossificans progressiva (FOP). Dorsomorphin analogues, first identified in zebrafish, remain the only BMP inhibitor chemotype reported to date. By screening an assay panel of 250 recombinant human kinases we identified a highly selective 2-aminopyridine-based inhibitor K02288 with *in vitro* activity against ALK2 at low nanomolar concentrations similar to the current lead compound LDN-193189. K02288 specifically inhibited the BMP-induced Smad pathway without affecting TGF-β signaling and induced dorsalization of zebrafish embryos. Comparison of the crystal structures of ALK2 with K02288 and LDN-193189 revealed additional contacts in the K02288 complex affording improved shape complementarity and identified the exposed phenol group for further optimization of pharmacokinetics. The discovery of a new chemical series provides an independent pharmacological tool to investigate BMP signaling and offers multiple opportunities for pre-clinical development.

## Introduction

Members of the transforming growth factor-beta (TGF-β) superfamily bind transmembrane receptor serine/threonine kinases to activate Smad and non-Smad pathways for the control of normal development and tissue repair [1], [2]. Ligand binding induces type II receptor phosphorylation of associated type I receptors, leading to Smad recruitment and phosphorylation by the type I receptor [3], [4]. The receptor-associated Smads (R-Smads) subsequently assemble with co-Smad4 for nuclear transport and transcriptional activation [5].

Small molecule inhibitors of the type I receptors (also known as activin receptor-like kinases, ALK1-7) have proved to be valuable pharmacological tools to characterize TGF-β and BMP pathways in signaling, as well as stem cell biology [6], [7]. TGF-β inhibitors such as SB-431542 inhibit Smad2/3 phosphorylation by ALK4, ALK5 and ALK7, as well as non-classical Smad1/5 phosphorylation by ALK5 [8]. Conversely, inhibitors of BMP signaling have recently been described that specifically inhibit Smad1/5/8 phosphorylation by ALK1, ALK2, ALK3 and ALK6 [9], [10], [11]. Notably, these molecules have shown efficacy in a variety of disease models, including chronic anemia [12], [13], [14], prostate cancer [15], muscle wasting [16], heterotopic ossification [17], atherosclerosis and vascular calcification [18], [19].

While specific TGF-β inhibitors have been developed over many years [20], BMP inhibitor development remains at an early stage [6]. New leads in this target area are desirable for several reasons. First, current work follows a single high throughput screen performed in the zebrafish system [9]. Second, independent tool compounds are preferred for functional validation, whereas the screening hit dorsomorphin [9], and derivatives DMH1 [10] and LDN-193189 [11], [17], share the same pyrazolo[1,5-a]pyrimidine scaffold. Third, more selective compounds are needed to minimize unwanted off-target effects [7].

Most importantly, there is an urgent need for selective ALK2 inhibitors to treat the debilitating bone disorder fibrodysplasia ossificans progressiva (FOP) [21]. FOP sufferers carry a gain of function mutation in the intracellular domain of ALK2, resulting in episodic bone formation in skeletal muscle and connective tissue that ultimately renders movement impossible [22]. Trauma and surgery only accelerate the condition, while biological inhibitors lacking cell penetrance are ineffective [23].

As an alternative but complementary strategy to phenotypic screens, we used direct screening of recombinant human kinases to identify new inhibitor leads against ALK2. We report a novel BMP inhibitor scaffold, comprising a 2-aminopyridine core and a trimethoxyphenyl specificity group, which is both potent and selective. The identified inhibitor K02288 provides a new pharmacological tool to investigate the diversity of BMP signaling in both normal and pathobiology.

## Results

### Identification of a Novel 2-aminopyridine Inhibitor of ALK2

To identify new potent and selective inhibitors of ALK2 we screened a kinase-directed library of 2000 compounds, including known biologically active molecules as well as novel chemotypes, against a panel of 80 purified human kinases using differential scanning fluorimetry (DSF) in a 96-well plate format [24], [25]. In this fluorescence-based thermal shift assay, compound binding to the native protein is observed as a relative increase in the protein’s melting temperature (Tm shift), proportional to the binding affinity. Typically for a protein kinase in the presence of 10 µM compound a Tm shift of 4°C corresponds to *K*_D_ of 1 µM, whereas a Tm shift above 8°C reflects a *K*_D_ of 100 nM or less [26].

**Figure 1:**
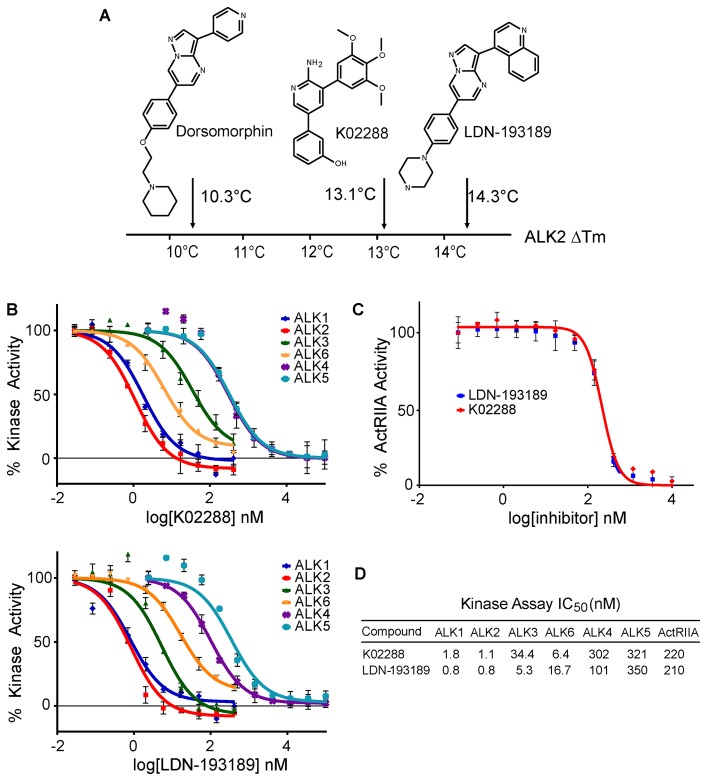
Identification of a novel 2-aminopyridine inhibitor of ALK2. (A) Schematic summary of a thermal shift assay screen using recombinant ALK2 kinase domain. A novel 2-aminopyridine hit K02288 was identified with an affinity for ALK2 intermediate between dorsomorphin and LDN-193189. Complete screening data are shown in supplemental Table S1. (B) *In vitro* kinase assays showed K02288 specificity for ALK1,2,3,6 over ALK4,5. IC_50_ measurements were performed in triplicate at the Km value of ATP. (C) ActRIIA kinase inhibition was determined using the Kinase-Glo® assay (Promega). IC_50_ measurements were performed in duplicate at the Km value of ATP. (D) Summary of IC_50_ measurements in all experiments.

Screening of human ALK2 was performed on the kinase domain residues 201–499 including the activating mutation Q207D. The known screening hit dorsomorphin produced a reference Tm shift of 10.3°C, consistent with its reported *in vitro* IC_50_ of 50 nM [6] (Figure 1A). A novel hit compound K02288 (3-[6-amino-5-(3,4,5-trimethoxy-phenyl)-pyridin-3-yl]-phenol) was identified containing a 2-aminopyridine scaffold that produced a significantly higher Tm shift of 13.1°C, suggestive of an improved affinity (Figure 1A). In the screen, a similarly high Tm shift (14.3°C) was observed only for the optimized dorsomorphin derivative LDN-193189 (Figure 1A). Importantly, K02288 was highly selective against the screening panel, showing a Tm shift greater than 8°C only for the homologous kinases ALK1-6 and ActRIIA (supplemental Table S1; values were again intermediate to those of dorsomorphin and LDN-193189). The screen also confirmed the binding of LDN-193189 to AMPKα2, as well as the more promiscuous binding of dorsomorphin (supplemental Table S1).

To confirm K02288 as a direct inhibitor of the activin receptor-like kinases we compared the activity of this hit compound against that of LDN-193189 in an *in vitro* kinase assay (Figures 1B and 1D). K02288 and LDN-193189 were most potent against the type I BMP receptors ALK1 and ALK2 (IC_50_s in the 1–2 nM range), which share 79% sequence identity within their kinase domains. Inhibition of other type I BMP receptors was slightly weaker (ALK3 and ALK6, IC_50_s of 5–34 nM). Both inhibitors also displayed an approximately 300-fold selectivity for ALK2 over the TGF-β receptor ALK5 (K02288 IC_50_ = 321 nM). Interestingly, K02288 further demonstrated an improved selectivity against ALK4 (IC_50_ = 302 nM). Finally, some weak inhibition of the type II BMP receptor ActRIIA was confirmed for both compounds, consistent with the thermal shift assay (IC_50_s of 210–220 nM, Figures 1C and 1D).

### Kinome-wide Selectivity

**Figure 2:**
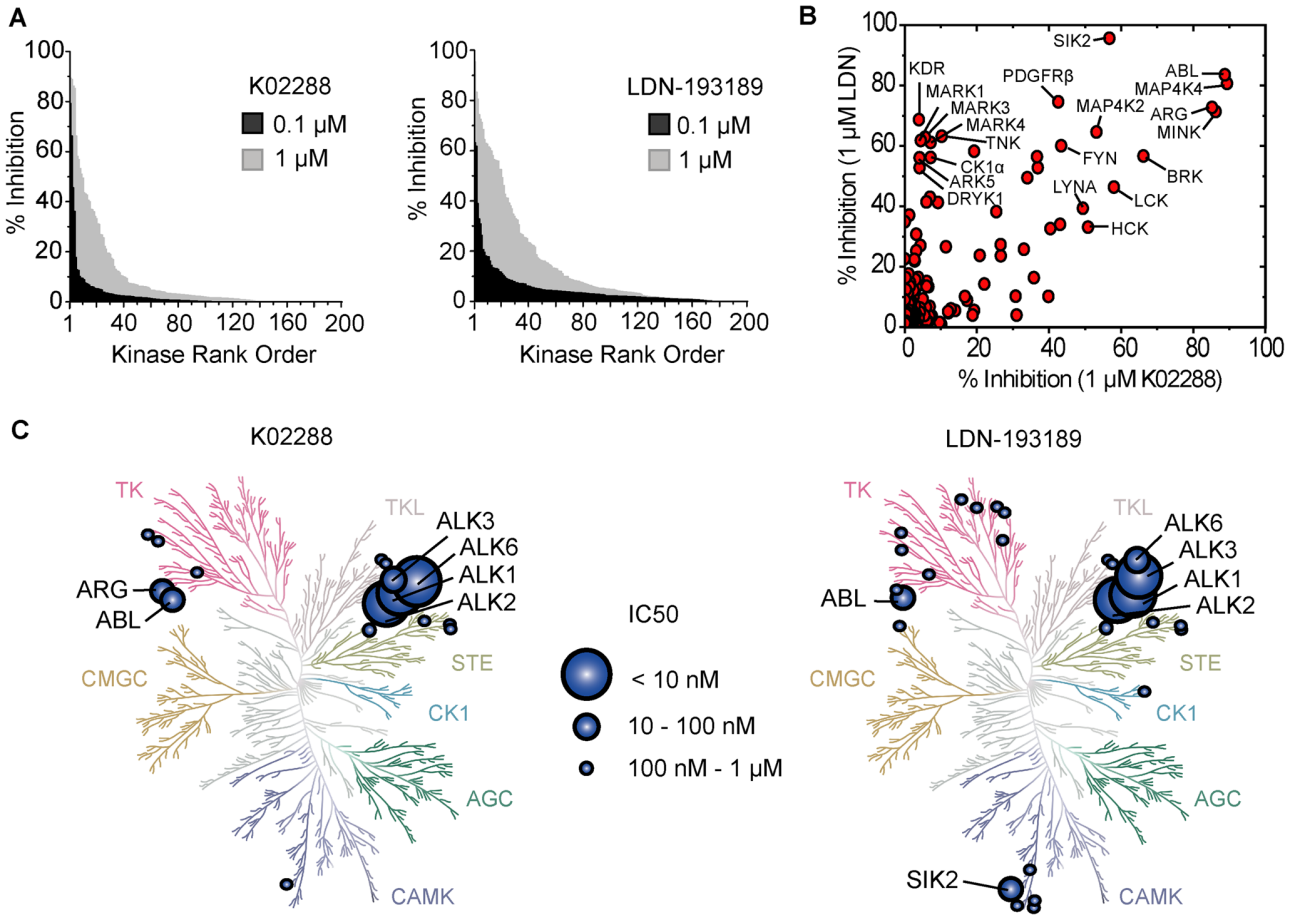
Kinome-wide selectivity of K02288 and LDN-193189. (A) Some 200 human kinases were individually ranked according to their enzymatic inhibition by K02288 or LDN-193189 present at 0.1 or 1 µM concentration (screening performed by Nanosyn). Complete screening data are shown in supplemental Table S2. (B) Percent inhibition values for each kinase in the presence of 1 µM K02288 were plotted against those using 1 µM LDN-193189. There is little correlation between those kinases inhibited by K02288 and by LDN-193189. Overall, fewer kinases were inhibited by K02288. (C) Kinome tree visualization of inhibitor profiling showing the appearance of target family clusters (illustration reproduced courtesy of Cell Signaling Technology, Inc.; www.cellsignal.com).

Enzymatic screening was extended to a further panel of 200 human kinases to evaluate the kinome-wide selectivity of K02288 and LDN-193189. Percentage inhibition was determined for each kinase at both 0.1 µM and 1 µM inhibitor concentration (supplemental Table S2). Overall, the selectivity profile of K02288 was more favorable than LDN-193189 (Figure 2A). K02288 showed >50% inhibition against only ABL and ARG (ABL2) at 0.1 µM inhibitor concentration, and only a further 6 kinases at 1 µM. In comparison, 1 µM LDN-193189 showed >50% inhibition against 21 kinases (10%), although only ABL and SIK2 were similarly inhibited at 0.1 µM. We were also interested to examine the extent to which the activities of K02288 and LDN-193189 were overlapping and thus the novelty of the K02288 chemical probe. We therefore plotted the percentage kinase inhibition values determined with 1 µM K02288 against those for 1 µM LDN-193189 to check for any correlation. As shown in Figure 2B, relatively few kinases, like ABL, were observed close to the diagonal, where activities are equivalent, indicating that the divergent inhibitor scaffolds yielded distinct selectivity profiles. Strikingly, a number of kinases, including KDR (VEGFR2), were inhibited exclusively by LDN-193189, but not by K02288 (Figure 2B). When visualized on a kinase phylogenetic tree, these were primarily clustered within the receptor tyrosine kinases (TKs) as well as the calmodulin-dependent kinases (CAMKs) (Figure 2C).

### Structural Basis for Inhibitor Binding

**Figure 3:**
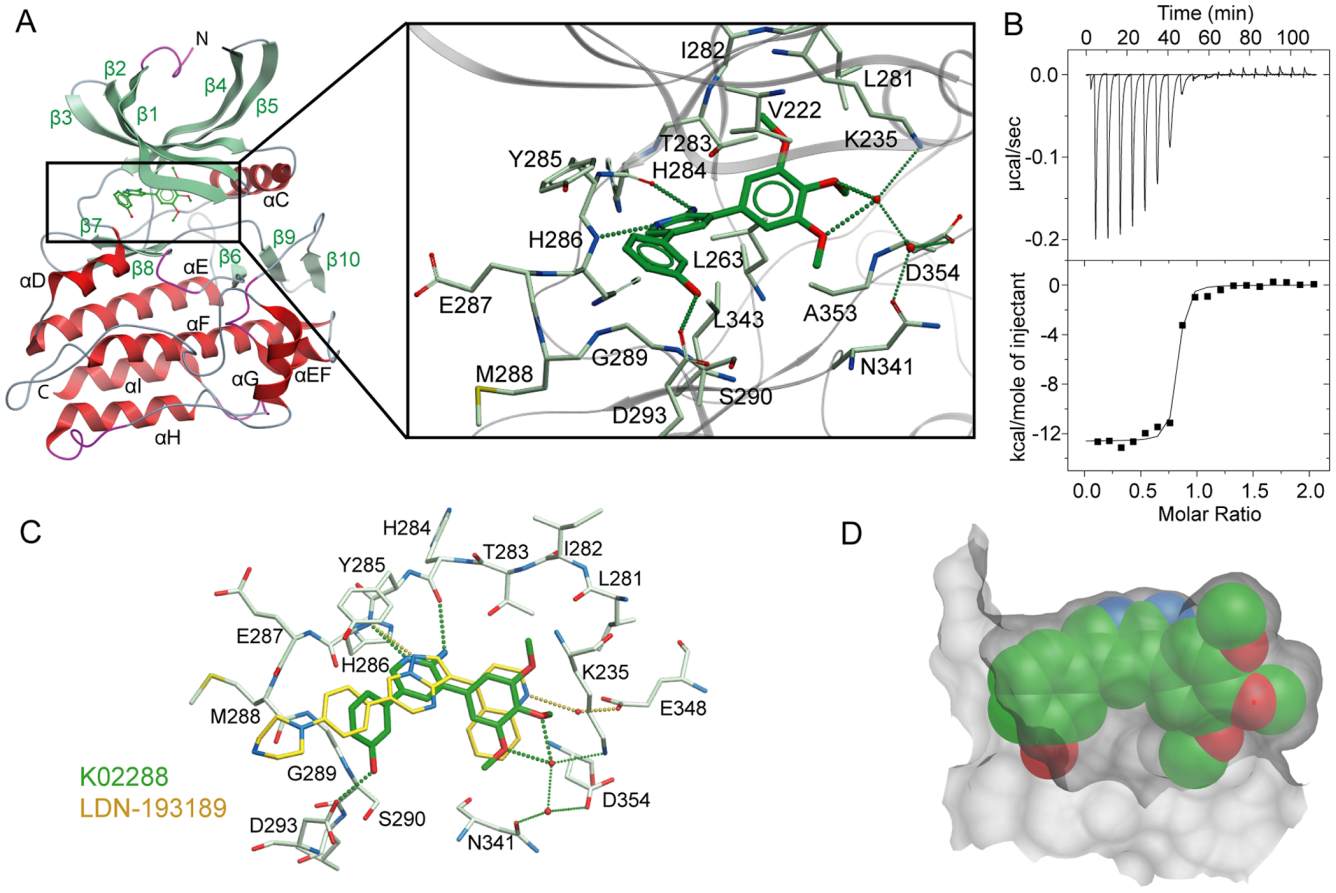
Structural basis for inhibitor binding to ALK2. (A) Structure of the ALK2-K02288 complex. Inset shows the interactions of K02288 in the ATP pocket. Hydrogen bonds are shown as dotted lines. (B) ITC measurements showed ALK2 bound K02288 with KD = 7.9 nM. (C) Comparison of the binding modes of K02288 (green) and LDN-193189 (yellow) in ALK2. Both inhibitors formed hydrogen bonds to the hinge residue H286, but other interactions were divergent, including water-mediated hydrogen bonds to K235 and E248, respectively. (D) A spacefill representation of K02288 shown against a surface mesh view of the ATP pocket highlights the shape complementarity in ALK2.

To define the mode of action of K02288 we determined its structure in complex with the kinase domain of ALK2 refined at 2.15 Å resolution (Figure 3A). The entire chain was traced from residues 202–499, including parts of the L45 loop and activation loop (A-loop) that were previously disordered in the structure of the ALK2-FKBP12 complex [27] (data collection and refinement statistics are provided in Table 1). Interestingly, the structure of the kinase domain was essentially unchanged by the loss of the GS domain, demonstrating that its inactive conformation was relatively stable (supplemental Figure S1). In particular, the ATP pocket remained occluded by the inhibitory conformations of the A-loop and αC helix, which were stabilized by the hydrogen bond interactions of R375 (A-loop) with S244 (αC), D336 (catalytic loop HRD motif) and D354 (A-loop DLG motif).

**Table 1:** Diffraction data and refinement statistics.

PDB accession code	3MTF	3Q4U
***Data collection***		
Beamline	Diamond I04	Diamond IO3
Wavelength (Å)	0.9762	0.9763
Resolution^a^ (Å)	41.85−2.15 (2.27−2.15)	41.88−1.82 (1.92−1.82)
Spacegroup	*P* 2_1_ 2_1_ 2	*P* 2_1_
Cell dimensions	*a* = 83.7, *b* = 138.3, *c* = 59.9 Å	*a* = 83.6, *b* = 98.7, *c* = 83.9 Å
	α = β = γ = 90.0°	α = γ = 90.0°; β = 117.4°
No. unique reflections^a^	37,445 (4,945)	107,198 (15,312)
Completeness^a^ (%)	97.3 (90.5)	99.2 (97.8)
I/σI^a^	10.5 (2.9)	11.6 (2.0)
R_merge_ a (%)	15.5 (63.8)	8.6 (73.4)
Redundancy^a^	7.9 (5.3)	4.3 (4.2)
***Refinement***		
Ligands	K02288	LDN-193189
No. refinement atoms (P/L/O)^b^	4,724/52/497	9,767/124/1,243
R_fact_ (%)	18.2	16.3
R_free_ (%)	24.4	21.9
B_f_ (P/L/O)^b^ (Å^2^)	25/17/33	28/23/37
rms deviation bond^c^ (Å)	0.016	0.016
rms deviation angle^c^ (°)	1.6	1.6
***Molprobity***		
Ramachandran favour	97.6%	97.8%
Ramachandran allowed	99.7%	100%

a Values in brackets show the statistics for the highest resolution shells.

b P/L/O indicate protein, ligand molecules presented in the active sites, and other (water and solvent molecules), respectively.

c rms indicates root-mean-square.

The 2-aminopyridine of K02288 was bound to the kinase hinge region in an ATP-mimetic fashion with two hydrogen bonds to H284 and H286, respectively (Figure 3A). The large trimethoxyphenyl substituent occupied the central pocket area sandwiched between the hydrophobic residues V222, L263, L343 and A353. Two of the three methoxy groups additionally formed a water-mediated hydrogen bond to the catalytic lysine (K235). The solvent channel was occupied by the phenol group, which formed an additional hydrogen bond with D293 (Figure 3A). The high affinity interaction of the inhibitor was confirmed by isothermal titration calorimetry (ITC), which indicated a *K*_D_ of 7.9 nM, close to the limit of measurement (Figure 3B).

We subsequently solved the ALK2 co-structure with LDN-193189 refined at 1.8 Å to compare its interaction (Figure 3C). The pyrazolo[1,5-a]pyrimidine core was bound similarly to the parent molecule dorsomorphin [27] with a single hydrogen bond to the hinge. Replacement of the 4-pyridine ring with 4-quinoline conserved the water-mediated hydrogen bond to E248 (αC helix), whilst providing improved potency through increased hydrophobic interaction. The piperazine substituent, selected for metabolic stability [11], was exposed to solvent. Superposition of the K02288 and LDN-193189 co-structures revealed a slight difference in their hinge-binding orientations allowing the trimethoxyphenyl of K02288 to adopt a similar position to the 4-quinoline (Figure 3C). Notably, the three methoxy substituents were able to extend more deeply towards the recesses of the pocket periphery, potentially contributing to the high selectivity of K02288. Indeed, the exquisite shape complementarity of this inhibitor scaffold was revealed by a ligand spacefill model and a surface skin representation of ALK2 (Figure 3D).

### K02288 Selectively Inhibits the BMP-Smad Pathway

**Figure 4:**
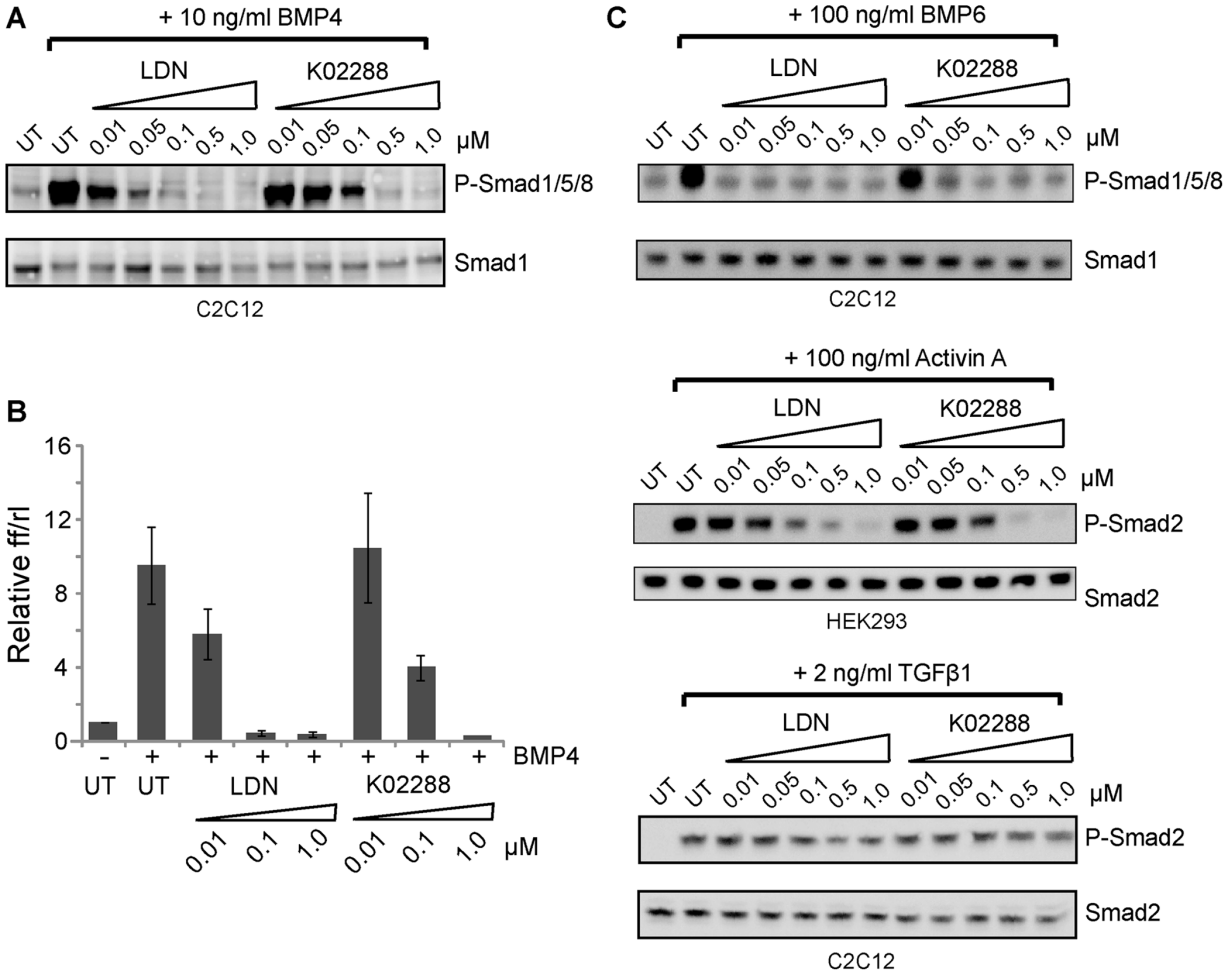
K02288 selectively inhibits BMP signaling. (A) K02288 and LDN-193189 inhibited BMP4 induced Smad1/5/8 phosphorylation in C2C12 cells. Phosphorylated Smads and total Smads were detected by Western blot. (B) BRE-luciferase assay in C2C12 cells showing the dose dependent inhibition of the BMP4 response. Cells were treated with LDN-193198 or K02288 at the indicated concentrations prior to BMP4 stimulation. Y-axis displays the ratio of Firefly/Renilla activity from three independent experiments each performed in triplicate ± S.E.M. (C) K02288 and LDN-193189 potently inhibited BMP6-induced Smad1/5/8 phosphorylation in C2C12 cells, but had no effect on TGF-β-induced phosphorylation of Smad2. Activin A-induced P-Smad2 in HEK293 cells was weakly inhibited by K02288 and LDN-193189.

The potent and selective activity of K02288 against ALK2 and related BMP receptors led us to explore the potential utility of K02288 as a chemical probe for BMP signaling in cells. The cell penetrance and activity of the compound were first confirmed using C2C12 cells and the ligand BMP4. Stimulation with BMP4 led to a robust phosphorylation of Smad1/5/8 that was reduced by K02288 in a dose dependent manner with an apparent IC_50_ of 100 nM (Figure 4A). Similar results were observed using a BMP response element (BRE)-luciferase reporter assay (Figure 4B). In both assays, the K02288 activity was 10-fold weaker than the control compound LDN-193189 (IC_50_ = 10 nM), suggesting that the K02288 scaffold should be optimized further for its cellular and metabolic stability.

We then tested the activity of both compounds against other ligands in the TGF-β superfamily to corroborate their specificity (Figure 4C). Indeed, the specificity of K02288 and LDN-193189 was remarkably similar, consistent with the thermal shift and *in vitro* kinase assay data. As expected, both compounds exhibited the highest activity against BMP6, a preferred ligand for ALK2 [28], with 50 nM K02288 giving near complete inhibition of Smad1/5/8 phosphorylation (Figure 4C). Neither compound inhibited TGF-β–induced phosphorylation of Smad2, in agreement with their observed specificity for ALK2 over ALK5 (Figure 4C). Activin A–induced phosphorylation of Smad2 was not observed in C2C12 cells and was tested instead in HEK293 cells. Interestingly, both compounds showed some weaker inhibition of this pathway, with near complete inhibition of Smad2 phosphorylation by 0.5 µM K02288 or LDN-193189 (Figure 4C). This activity likely reflects some weak inhibition of ALK4 or the type II activin receptors.

### K02288 Induces Dorsalization of Zebrafish Embryos

**Figure 5:**
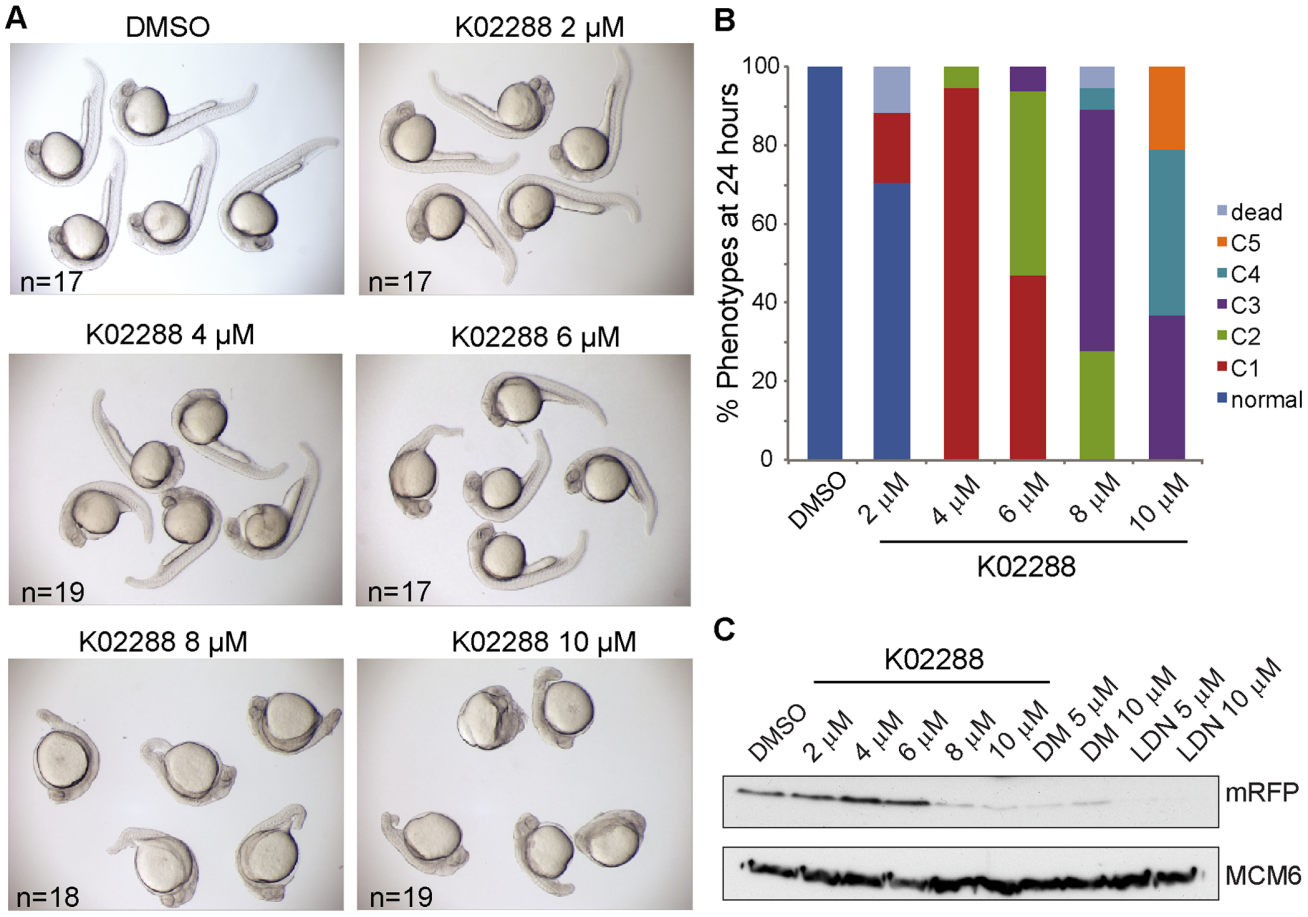
K02288 induces dorsalization of zebrafish embryos. (A) Brightfield photographs of 26 hours old *Tg(BRE:mRFP)* transgenic embryos treated with DMSO or varying doses of K02288 from the 8- to 16-cell stage. Severity of the dorsalization correlated with the dose of K02288. Very strong dorsalized phenotypes were observed with 8–10 µM K02288. (B) The phenotypes of the embryos shown in A were classified according to Kishimoto et al. [49]. (C) Western blot for mRFP in extracts prepared from *Tg(BRE:mRFP)* embryos treated in a parallel experiment. Loss of mRFP protein was evident at 8–10 µM K02288. As a control, the effects of dorsomorphin (DM) and LDN-193189 (LDN) on mRFP expression are also shown. Protein loading control is shown with the MCM6 blot.

To further validate K02288 as a pharmacological tool we tested its effects on *Tg(BRE:mRFP)* transgenic zebrafish embryos, which expressed monomeric red fluorescent protein (mRFP) under the control of a BMP response element [29]. Intact BMP signaling has been shown to be essential for proper specification of tissue progenitors across the dorsoventral axis [30]. Treatment with K02288 induced a dorsalized phenotype in a dose dependent manner (Figure 5A), as shown previously for dorsomorphin [9]. A severely dorsalized phenotype was observed at 8–10 µM concentration (Figure 5B) correlating with the loss of expression of mRFP protein (Figure 5C).

**Figure 6:**
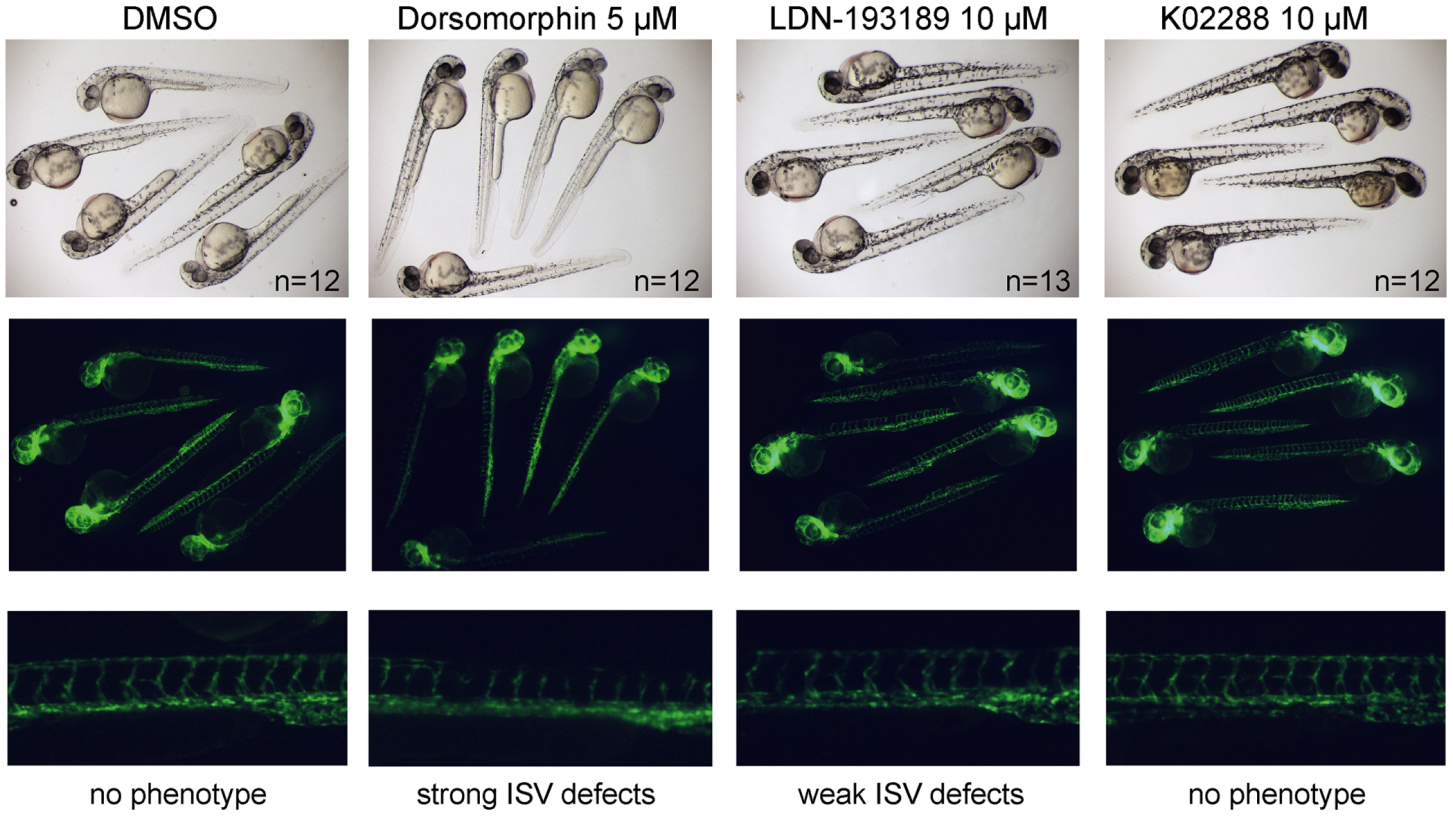
K02288 does not inhibit vasculature development. (Top panels) Brightfield photographs of 48 hours old *Tg*(*fli1a*:*eGFP)* embryos treated with DMSO or chemical inhibitors from 12 hours post fertilization. Embryos were manually dechorionated after bud stage before treatment. (Center panels) Same view under UV light for visualization of eGFP expression in the vasculature. Dorsomorphin and LDN-193189 treatment resulted in intersomitic vessel (ISV) formation defects, consistent with their known inhibition of VEGF signaling. (Lower panels) Higher magnification views of representative embryos and phenotype summary. The most severe phenotypes were observed with dorsomorphin. No effects on ISV formation were observed with K02288 treatment.

Finally, at 12 hours post fertilization (hpf) different inhibitors were administered to *Tg(fli1a:eGFP)* zebrafish embryos to compare their off-target effects on vascular development (Figure 6). At 48 hpf, embryos treated with 10 µM dorsomorphin or LDN-193189 exhibited defects in intersomitic vessel (ISV) formation, consistent with their known inhibition of VEGF signaling (reported KDR IC_50_s of 25 nM and 215 nM, respectively [10]). In contrast, no ISV defects were observed using 10–20 µM K02288 (Figure 6 and data not shown), as predicted by the screening data showing no inhibition of KDR (supplemental Table S2).

## Discussion

**Figure 7:**
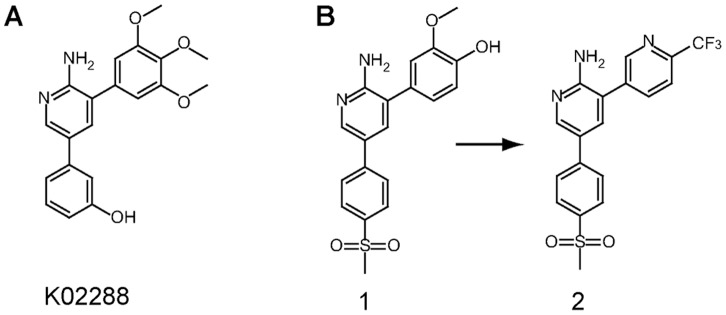
The K02288 scaffold is similar to an anti-malarial compound. (A) The 2-aminopyridine scaffold of K02288. (B) A similar initial hit **1** was identified in a recent anti-malarial screen and optimized to the more divergent lead **2** in development of the pre-clinical candidate MMV390048 [34].

The development of selective small molecule inhibitors of protein kinases presents a major challenge due to the high sequence conservation of the ATP pocket. Here we report a novel 2-aminopyridine inhibitor K02288 with potent and selective activity against type I BMP receptor kinases. The 2-aminopyridine group is an ATP-mimetic that binds the kinase hinge region through two conserved hydrogen bonds. Importantly, selective kinase inhibitors with this scaffold have been identified previously [31], [32]. The most advanced molecule crizotinib, which targets the receptor tyrosine kinases ALK and c-MET, has been approved by the US Food and Drug Administration for the treatment of patients with ELM4-ALK positive non-small-cell lung carcinoma [33]. Moreover, 3,5-diaryl-2-aminopyridines resembling K02288 were recently discovered as anti-malarials [34] (Figure 7), although we observed no effect of the lead compound on BMP or TGF-β signaling (data not shown).

K02288 exhibits remarkable potency for a low molecular weight screening hit, both in enzymatic assays (IC_50_s in the range 1–2 nM against ALK1 and ALK2) and in C2C12 cells (IC_50_<50 nM against BMP6 signaling). In comparison, the one previous screening hit dorsomorphin displayed IC_50_s of 50 nM in enzymatic assays [6] and ∼0.5 µM in C2C12 cells [35]. These activities were improved significantly following further chemistry to yield the lead derivative LDN-193189 [11], [17]. Similar optimization of the cellular and *in vivo* activity of K02288 would be beneficial to fully exploit its significant selectivity and could be achieved by replacement of the potentially vulnerable phenol moiety. In the crystal structure of the ALK2-K02288 complex this group bound to the exposed solvent channel where substitutions are likely to be well tolerated.

The discovery of diverse BMP inhibitor scaffolds establishes a repertoire of pharmacological tool compounds for cross-validation in investigations of cellular signaling. Moreover, the application of multiple orthogonal chemotypes may help to discern whether a particular toxicological liability is a class-wide pharmacological phenomenon due to ALK2 inhibition or the result of a chemotype specific off-target effect. The novel inhibitor K02288 provides an exciting new starting point for further chemistry with potential therapeutic applications in stem cell engineering, as well as in disease models of anemia, musculoskeletal dysplasia and cancer.

## Materials and Methods

### Protein Expression and Purification

The human ALK2 kinase domain, residues 201–499 including the activating mutation Q207D, was subcloned into the vector pFB-LIC-Bse. Baculoviral expression was performed in Sf9 insect cells at 27°C, shaking at 110 rpm. Cells were harvested at 48 hours post infection and resuspended in 50 mM HEPES pH 7.5, 500 mM NaCl, 5 mM imidazole, 5% glycerol, 0.1 mM TCEP, supplemented with protease inhibitor set V (Calbiochem). Cells were lysed using a C5 high pressure homogenizer (Emulsiflex) and the insoluble material excluded by centrifugation at 21,000 rpm. Nucleic acids were removed on a DEAE-cellulose column before purification of the N-terminally His-tagged ALK2 protein by Ni-affinity chromatography. The eluted protein was cleaved with TEV protease and further purified by size exclusion chromatography using a S200 HiLoad 16/60 Superdex column. A final clean up step was performed by reverse purification on a Ni-sepharose column and the buffer adjusted to 50 mM HEPES pH 7.5, 300 mM NaCl, 10 mM DTT, 50 mM L-arginine and 50 mM L-glutamate. Excess protein was flash frozen and stored at −80°C.

### Differential Scanning Fluorimetry (DSF)

Thermal melting experiments were performed using a Real Time PCR machine Mx3005p (Stratagene) with a protein concentration of 1–2 µM and 10 µM inhibitor as described previously [24], [25]. A kinase-directed compound set, including K02288, was purchased from Biofocus (DPI). Dorsomorphin and other known biologically active kinase inhibitors were purchased from Calbiochem. LDN-193189 was prepared as described previously [11]. Recombinant human kinases for DSF screening were prepared by SGC using published methods [26], [36].

### *In vitro* Kinase Assay for ALK1-6

Kinase reactions for ALK1-6 were performed at room temperature for 45 minutes in 96-well plates mixing 2.5 nM kinase (Invitrogen), 0.5 mg/mL dephosphorylated casein (Sigma), 6 µM ATP (Sigma), 0.05 µCi/µL [γ-^32^P]ATP (Perkin Elmer), 10 mM MnCl_2_ and 0.2% BSA in kinase buffer (Cell Signaling). Inhibitors were added at concentrations between 0 and 10 µM in kinase reaction buffer and tested in triplicate. Reactions were quenched with phosphoric acid, bound to 96-well P81 phosphocellulose filter plates (Millipore) and assayed with Microscint 20 scintillation fluid (Perkin Elmer) using a Spectramax L luminometer (Molecular Devices). Data were normalized to untreated controls at 100% enzyme activity and negative controls subtracted as background. IC_50_ values were calculated using GraphPad (Prism software).

### Kinase-Glo® Assay

A kinase assay for ActRIIA (ACVR2) was performed using Kinase-Glo® (Promega) as per manufacturer’s instructions. Briefly, the following were mixed and reacted at room temperature for 3 hours in a 96-well plate at a final volume of 100 µL, 10 nM kinase (∼EC_50_ at 2 hr), 0.5 mg/mL dephosphorylated casein (Sigma), 10 µM ATP (Promega), 10 mM MnCl_2_ and 0.2% BSA in kinase buffer (Cell Signaling). Inhibitors were added at concentrations between 0 and 10 µM in kinase reaction buffer and tested in duplicate. At 15 min, 30 min, 1 hr, 2 hr, and 3 hr 20 µL aliquots of the reaction mixture was transferred to a 384-well plate and 20 µL of Kinase-Glo® was added and allowed to rest for 10 min to quench the reaction and produce light which was measured using a Spectramax L luminometer (Molecular Devices). The 2 hr time point was within the linear portion of the reaction and was used for calculations due to favourable signal-to-noise ratio and was consistent with earlier time points. Data were normalized to untreated controls at 100% enzyme activity and negative controls subtracted as background. IC_50_ values were calculated using GraphPad Prism (GraphPad Software, Inc).

### Kinase-wide Selectivity Profiling

Inhibitor selectivity profiling against 200 human kinases at 0.1 and 1 µM inhibitor concentration was performed by Nanosyn (www.nanosyn.com).

### Crystallization, Data Collection and Structure Determination

Crystallization was performed using the sitting drop vapour diffusion method at 20°C. Viable crystals of ALK2 in complex with LDN-193189 grew in a 150 nL drop mixing 10 mg/mL protein, pre-incubated with 1 mM compound, with a reservoir solution containing 20% PEG3350, 0.2 M ammonium citrate dibasic pH 5.0 at 1∶1 volume ratio. Crystals were transferred into a cryo-protective solution prepared from the mother liquor supplemented with 20% ethylene glycol. Diffraction data were collected at Diamond Light Source, beamline I03. For the K02288 complex, frozen ALK2 protein was thawed, purified from aggregates on a S200 HiLoad 16/60 Superdex column, and concentrated to 7 mg/mL. Viable crystals of ALK2 in complex with K02288 grew in a 150 nL drop mixing the protein, pre-incubated with 1 mM compound, with a reservoir solution containing 1.6 M Na/K phosphate, 0.1 M HEPES pH 7.5 at 1∶2 volume ratio. Crystals were transferred into a cryo-protective solution prepared from the mother liquor supplemented with 25% ethylene glycol. Diffraction data were collected at Diamond Light Source, beamline I04. Data were processed and scaled with MOSFLM and SCALA from CCP4 suite [37], [38]. Structures were solved by molecular replacement using PHASER [39] and the structure of ALK2 from the ALK2-FKBP12-dorsomorphin complex [27] as a search model. Subsequent manual model building was performed using COOT [40] alternated with refinement in REFMAC [41]. TLS-restrained refinement was applied in the latter cycles using the input thermal motion parameters determined by the TLSMD server [42]. The final models were verified for geometry correctness with MOLPROBITY [43]. Data collection and refinement statistics are summarized in Table 1.

### Phospho-Smad Determination

C2C12 and HEK293 cells were grown in DMEM supplemented with 10% FCS (PAA) and seeded at a density of 1 x 10^5^ cells per well in 6-well plates. The next day the cells were starved in DMEM containing 1% FCS for 5 hours. Inhibitors were added at the indicated concentrations for 30 minutes followed by 10 ng/mL BMP4 (Peprotech), 100 ng/mL BMP6 (Peprotech), 100 ng/mL Activin A (Peprotech) or 2 ng/mL TGF-β1 (Cell Signaling) for 1 hour. Cells were washed in PBS and lysed on ice for 30 minutes in lysis buffer (20 mM Tris-HCl pH 7.5, 150 mM NaCl, 1% Triton X-100, 25 mM NaF, 25 mM Na β-glycerophosphate, 2 mM Na_3_VO_4_) containing protease inhibitors (Roche). Protein concentration in the clarified lysate was determined by Bradford Assay (Pierce) and 15 µg run on 4–12% Bis-Tris gel (Biorad). The protein was transferred onto nitrocellulose membrane (GE Healthcare) and probed with the relevant antibody at 4°C overnight (Cell Signaling: anti-Smad 1 (#9743), anti-P-Smad 1/5/8 (#9511), anti-Smad 2 (#5339), anti-P-Smad 2 (#3101). After subsequent incubation with HRP-conjugated anti-rabbit antibody, protein bands were detected using ECL (Pierce) and an LAS4000 image reader. Cells were purchased originally from the European Collection of Cell Cultures (ECACC) available through Sigma.

### Dual Luciferase Reporter Assay

C2C12 cells were co-transfected using lipofectamine 2000 with BRE-luciferase [44] and Renilla Luciferase pRLTK (Promega) following the manufacturer’s instructions. 16 hours post transfection cells were starved in DMEM containing 1% FCS for 5 hours. Inhibitors were added to triplicate wells at the indicated concentrations for 30 minutes, followed by overnight stimulation with 10 ng/ml BMP4 (Peprotech). Luciferase activities were determined according to the Dual-Luciferase® Reporter Assay System (Promega) using Renilla for normalization of transfection efficiency.

### Fish Maintenance and Transgenic Lines

The zebrafish colony was maintained as described [45] and embryos were raised at 28.5°C. The *Tg(BRE:mRFP)* transgenic line (also called BRE-mRFP) has been described previously [29] and expresses mRFP in response to BMP signaling. The *Tg(fli1a:eGFP)* transgenic line has also been described and used to test the off-target effects of dorsomorphin and LDN-193189 on vasculature development [10], [46], [47].

### Chemical Inhibitor Treatment of Zebrafish

Inhibitor stocks were diluted in DMSO and then further diluted in fish water to give the required inhibitor concentrations. To minimize the amount of inhibitors used, experiments were performed in 24-well plates with approximately 20 embryos per well in a volume of 1 mL. The chemical treatment was applied by immersing 8- to 16-cell stage *Tg(BRE:mRFP)* embryos (dorsalization experiment) or 12 hours old *Tg(fli1a:eGFP)* embryos (ISV experiment) in the fish water supplemented with DMSO or the chemical inhibitors. For the ISV experiment, embryos were manually dechorionated after bud stage before treatment. Embryos were scored and photographed at 26 or 48 hours.

### Zebrafish Protein Extraction and Western Blotting

Ten non-dechorionated 26 hours old treated *Tg(BRE:mRFP)* embryos were snap frozen on dry ice and stored at −80°C. Protein extracts were prepared as described previously for *Xenopus* embryos [48]. Extraction buffer was freshly prepared. 10 µL per embryo of the protein extract buffer was used to lyse the embryos using a plastic pestle and a pellet pestle motor (Kontes). 40 µg (∼ 1 embryo equivalent) of protein extract was loaded per lane on a 12.5% SDS-PAGE gel. The rabbit anti-mRFP (Invitrogen, R10367) and the goat anti-MCM6 (Santa Cruz, sc-9843) antibodies were used for Western blots.

## Supporting Information

Figure S1 **The ALK2 kinase domain adopts an inactive conformation.** Superposition of the ALK2-K02288 structure (PDB 3MTF) and the ALK2-FKBP12-dorsomorphin structure (PDB 3H9R) reveals no structural change despite the absence of the GS domain and bound FKBP12. The kinase domain in ALK2 appears stable in an inactive conformation. In particular, the ATP pocket is occluded by the inhibitory conformations of the activation segment (including the β9-β10 hairpin) and the αC helix, which are stabilized by the hydrogen bond interactions of R375 (activation segment) with S244 (αC), D336 (catalytic loop HRD motif) and D354 (activation segment DLG motif).
(TIF)

Table S1 **Differential scanning fluorimetry screening against 80 recombinant human kinases.**
(PDF)

Table S2 **Kinome-wide selectivity profiling performed by Nanosyn (** www.nanosyn.com **).**
(PDF)
