# Supplementary figures and images for: A New Class of Small Molecule Inhibitor of BMP Signaling
Source: PLoS One. 2013 Apr 30;8(4):e62721. doi: 10.1371/journal.pone.0062721 (PMC3639963; doi:10.1371/journal.pone.0062721)

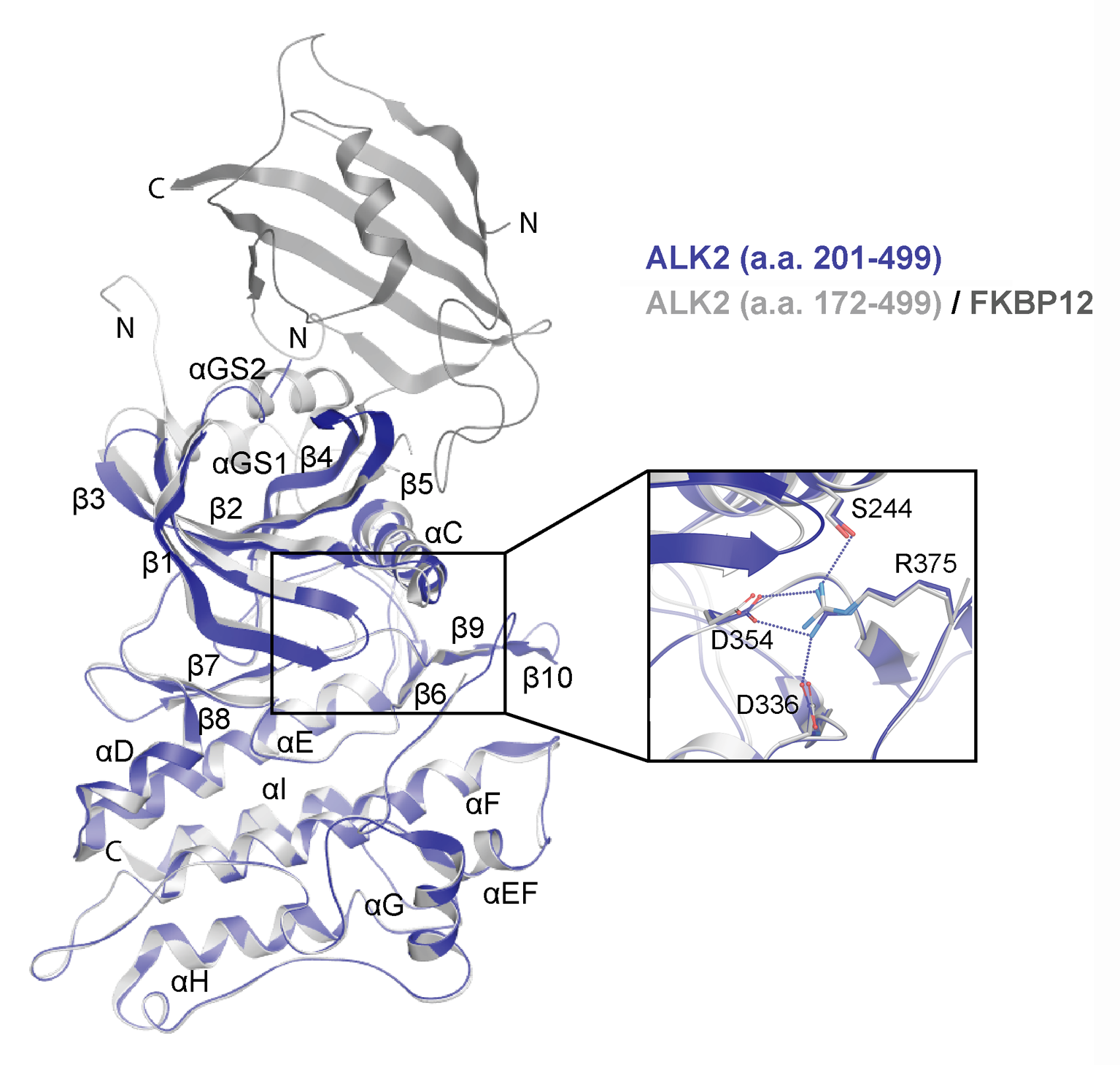

Supplement: Figure S1 — The ALK2 kinase domain adopts an inactive conformation. Superposition of the ALK2-K02288 structure (PDB 3MTF) and the ALK2-FKBP12-dorsomorphin structure (PDB 3H9R) reveals no structural change despite the absence of the GS domain and bound FKBP12. The kinase domain in ALK2 appears stable in an inactive conformation. In particular, the ATP pocket is occluded by the inhibitory conformations of the activation segment (including the β9-β10 hairpin) and the αC helix, which are stabilized by the hydrogen bond interactions of R375 (activation segment) with S244 (αC), D336 (catalytic loop HRD motif) and D354 (activation segment DLG motif). (TIF) [file pone.0062721.s001.tif]
