# Supplementary material for: A New Class of Small Molecule Inhibitor of BMP Signaling
Source: PLoS One. 2013 Apr 30;8(4):e62721. doi: 10.1371/journal.pone.0062721 (PMC3639963; doi:10.1371/journal.pone.0062721)
Supplement: Table S1 — Differential scanning fluorimetry screening against 80 recombinant human kinases. (PDF) [file pone.0062721.s002.pdf]

Table S1. Differential scanning fluorimetry screening against 80 recombinant human kinases.

Kinase	Tm shift (°C)		
	Dorsomorphin	LDN-193189	K02288
AAK1	3.8		0.5
ActRIIA	6.1	8.6	8.3
AKT3	0.5		0.5
ALK1	10.6	15.7	14.2
ALK2	10.3	14.3	13.1
ALK3		14.0	12.8
ALK4	6.1	11.0	8.2
ALK5	7.4	12.0	11.6
ALK6	12.4	17.1	13.9
AMPKα1	3.9	4.3	0.2
AMPKα2	7.1	8.3	1.2
BMPRII	1.5	-0.4	3.5
BMX	1.4		1.0
BRK	2.8		0.0
CAMK1δ	-0.3		-0.3
CDK2	2.5	0.7	0.1
CDK8	0.5		0.1
CDKL1	2.0		0.0
CHEK2	1.7	1.4	0.0
CK1γ1	0.4	0.3	0.7
CK1γ3	1.2		2.0
CK1ε	5.9		4.5
CK2α1	3.8		0.3
CLK1	8.3		-0.1
CLK2	5.0	3.3	0.0
CLK3	3.7	0.7	0.0
CLK4	8.7	5.3	0.3
DAPK3	1.2		0.2
DCAMKL1	0.3		0.1
DRAK1	9.0		1.0
DRAK2	10.0	2.1	0.6
DYRK1A	4.4		-0.9
DYRK2	5.1		0.3
ERK1	0.0	0.2	0.2
FES	0.5	0.4	0.4
GAK	4.3		6.9
GRK1	0.4	-0.7	0.0
GRK3	-0.1		0.1
GUCY2D	-0.1	-0.2	
Haspin	2.3	0.4	0.3

Kinase	Tm shift (°C)		
	Dorsomorphin	LDN-193189	K02288
JNK1	0.0	0.5	0.2
JNK2	0.4	-0.6	-0.1
LOK	2.5	3.1	2.1
MEK2	-0.1	2.1	2.4
MEK6	0.1		1.9
MPSK1	5.3	5.3	0.3
MRCKβ	0.0	0.6	0.4
MST3	4.6	7.9	0.3
MST4	0.7	0.8	0.2
MYLK4	4.3		1
NDR1	2.8		0.1
NEK1	0.1	0.2	0.2
NEK11	2	-0.3	0.3
OSR1	1.6	0.9	0.0
p38β	1.1		0.5
p38δ	0.0	-0.4	-0.2
PCTAIRE1	4.7		-1.8
PIM1	0.6	0.5	0.1
PIM2	0.6	-1.3	0.1
PIM3	0.6	-0.2	-0.2
PKCη	0.9		1.2
PKD2	0.1		0.2
PKD3	0.9		1.2
PKMYT1	0.1		-0.4
PKN1	2.3	2.3	0.5
PLK4	4.2	3.7	0.0
PRKX	2.9		0.2
PTK7	-1.2	-0.3	-1.3
RSK1	2.8	3.3	1.1
RSK4	2.7		1.4
SLK	1.6	1.2	1.0
SRPK2	0.2		0.2
STK33	4.2	6.5	1.1
TNIK	4.2	4.6	6.1
TOPK	0.3		-0.2
TTK	1.0		1.1
VRK1	0.4		0.5
VRK2	0.2	-0.2	0.6
YANK1	0.5	1.2	0.2
ZAK	0.5	2.7	-0.1
