# Supplementary material for: A New Class of Small Molecule Inhibitor of BMP Signaling
Source: PLoS One. 2013 Apr 30;8(4):e62721. doi: 10.1371/journal.pone.0062721 (PMC3639963; doi:10.1371/journal.pone.0062721)
Supplement: Table S2 — Kinome-wide selectivity profiling performed by Nanosyn ( www.nanosyn.com ). (PDF) [file pone.0062721.s003.pdf]

Table S2. Kinome-wide selectivity profiling performed by Nanosyn (www.nanosyn.com).

	Compound			
	K02288 (μM)		LDN-193189 (μM)	
Kinase	0.1	1	0.1	1
ABL1	79	89	62	84
AKT1	-2	-1	0.8	1.6
AKT2	-2	-1	1	1.8
AKT3	-2	1.2	0.2	1.5
ALK	-1	2.5	3.7	12
AMPKα1	-1	-0	5.2	23
ARG	66	85	33	73
ARK5	1.1	4.1	16	56
AURKA	-2	-1	4.2	23
AURKB	0.3	1.5	2.3	15
AURKC	-1	3.2	4.2	25
AXL	0.9	1.5	-4	1.1
BLK	3.2	27	3.9	24
BMX	0.5	19	0.2	4
BRAF	-0	12	3.5	5.2
BRK	13	66	13	57
BRSK1	2.3	-0	3	17
BRSK2	0.9	2.7	4.4	22
BTK	0.5	6.6	0.5	1.8
CAMK1δ	-2	-1	0.8	0.9
CAMK2α	-2	-2	-0	-8
CAMK2δ	-1	1.4	3.1	8.7
CAMK4	-1	-1	0.1	-0
CDK1/cycB	1	1.5	0.5	5
CDK2/cycA	-0	-0	1.2	4.1
CDK2/cycE	0.2	1.5	2.8	5.3
CDK3/cycE	1.6	-91	1.6	7
CDK4/cycD	0.1	1.3	0.8	8.3
CDK5/p35	0.3	1.9	1.4	8.5
CDK6/cycD3	1.7	2.8	7.2	22
CHEK1	-1	-2	2.9	0.3
CHEK2	-1	2.5	1.5	2
CK1α	-0	7.2	14	56
CK1γ1	-0	6.1	3.1	14
CK1γ2	-1	5.5	1.6	3.9
CK1γ3	2.7	19	2.3	5.5
CK2	6.2	2.8	0.7	4.2
CLK2	-0	0	2.3	35
CLK3	1.4	1.5	0.5	2.6
cRAF	-5	-8	0.6	0.1

	Compound			
	K02288 (μM)		LDN-193189 (μM)	
Kinase	0.1	1	0.1	1
CSK	3.2	13	3.3	6
DAPK1	-1	-1	1.5	0.4
DCAMKL2	-2	2.3	0.8	3.4
DDR2	-0	11	5.5	27
DYRK1A	1.9	4.1	10	53
DYRK1B	2.6	6	7.1	41
DYRK2	-1	-3	2.3	0.2
EGFR	2.4	12	1.5	4.8
EPHA2	-2	3	-2	-3
EPHA3	-6	4.3	2.2	6.3
EPHA4	-2	17	7.1	10
EPHB2	-0	21	8.4	24
EPHB3	-2	-1	5.6	4.3
EPHB4	-0	5.9	-1	6.6
ERBB2	-5	-3	2.1	2.1
ERBB4	-5	0.9	6.1	11
FER	0.8	7.5	2.8	3.7
FES	-1	-1	2.2	2.4
FGFR1	-1	-3	3.4	19
FGFR2	0.3	1.1	5	18
FGFR3	-2	0.2	5	16
FGFR4	4	-0	0.3	2.4
FGR	6.7	37	13	53
FLT1	0	-2	0	17
FLT3	-2	0.4	3.4	12
FLT4	0.6	1.4	3.4	13
FMS	6.1	33	3.7	26
FYN	10	43	18	60
GRK6	0.2	1.4	-0	0.2
GRK7	0.2	-3	1.8	0.8
GSK3α	0.6	2.1	3.2	1.1
GSK3β	0.5	1.6	2.7	2.1
HCK	8.9	51	9.7	33
HIPK1	1.7	3.1	0.6	4.4
HIPK4	0.8	4.2	-0	4.9
IGF1R	-3	-1	4.1	5.4
IKK-α	7.4	2.7	3	-2
IKK-β	-2	1.3	0.3	1.4
IKK-ε	-6	-2	7.5	7.4
INSR	-4	0.7	3.8	4.5

	Compound			
	K02288 (μM)		LDN-193189 (μM)	
Kinase	0.1	1	0.1	1
IRAK4	-1	0.3	0.7	1.9
IRR	-1	-1	1.9	2.2
ITK	-0	-0	0.9	3.6
JAK1	-0	0.4	2.7	4.2
JAK2	-0	-0	2	0.5
JAK3	5.6	2.3	-0	0.1
JNK2	0.9	-0	3.9	1.3
KDR	0.5	3.9	19	69
KIT	4	9.2	7	41
LCK	13	58	12	46
LOK	3.5	25	8.3	38
LRRK2	7.1	27	8.7	27
LTK	3.6	31	3.6	4
LYNA	9.7	49	9.1	39
LYNB	6	43	7.4	34
MAP4K2	18	53	21	65
MAP4K4	46	89	31	81
MAPK1	-3	9.9	2.6	1.4
MAPK3	-0	-1	1.9	-2
MAPKAPK2	-2	-1	0.7	-0
MAPKAPK3	-1	-2	1.6	0.5
MARK1	1.3	4.5	13	62
MARK3	2.6	5.6	11	63
MARK4	2.4	7.2	13	61
MEK1	-1	10	1.4	1
MER	5.1	40	4.4	10
MET	0.6	-2	6.2	8.4
MINK	36	86	26	71
MKNK1	0.7	1.3	7.1	37
MNK2	1.9	3.2	3.7	31
MRCKα	2	6.9	0.8	6.8
MRCKβ	0.6	2	-0	2.1
MSK1	-2	-1	1.1	1.3
MSK2	-1	1	0.9	0.8
MSSK1	1.8	-0	-3	1.5
MST1	-1	1.1	4	7.9
MST2	-2	-1	5.1	6
MST4	0.4	0	-1	-5
MUSK	1.2	-1	5.8	26
NEK1	-1	0.8	2.1	3.4

	Compound			
	K02288 (μM)		LDN-193189 (μM)	
Kinase	0.1	1	0.1	1
NEK2	-2	-2	2.8	1.2
NEK6	-2	-1	2.3	-1
NEK7	0.2	-3	7.9	1.7
NEK9	5.3	-2	1.9	6.6
P38α	-3	1.5	4.4	3.3
P38β	-0	9.7	0.4	1.5
P38γ	-1	-1	1.2	1.3
P38δ	-3	-3	1.8	0.5
P70S6KB1	-2	-1	1	0.6
PAK1	-0	0.6	4.3	4.9
PAK2	-1	-1	-0	-0
PAK3	-3	-2	2.9	1.8
PAK5	-1	-1	6.2	8.5
PAK6	0.8	1.9	8.8	5.1
PAR1Bα	2.3	7	6.9	43
PASK	1.1	0.6	1.9	1.4
PDGFRα	5.3	37	18	56
PDGFRβ	7.7	43	39	75
PDK1	-0	0.5	1.1	1.9
PHKγ1	1.8	4.4	5.2	27
PHKγ2	-3	-3	3.5	4.9
PI3-K-α	2.6	2	2.1	1.5
PI3-K-δ	0.8	2.5	-1	10
PI4-K-β	0.8	-4	3.5	5.8
PIM-1	0.2	1.5	-1	-1
PIM-2	0.9	2.3	-0	-0
PIM3	2.3	5.9	2	-1
PKA	0.3	6.1	1.4	2.1
PKCα	0.2	2.2	1	1.1
PKCβ1	-1	0.7	-2	0.2
PKCβ2	-0	-1	1	1.8
PKCγ	-4	-1	2.1	4.1
PKCη	-2	-4	2.2	-2
PKCθ	-0	0.1	1.4	1.3
PKCι	0.3	1.6	1.4	1.5
PLK1	-2	-2	0.8	1.3
PRAK	-0	-0	0.8	0.1
PRKD1	-0	6.1	4.6	15
PRKD2	0.6	6.6	4.5	13
PRKD3	-1	3.2	3.2	13

	Compound			
	K02288 (μM)		LDN-193189 (μM)	
Kinase	0.1	1	0.1	1
PRKG1	0.3	3.3	1	1.2
PRKG2	1	5	1.7	9.3
PRKX	-1	-1	0.2	1.2
PTK5	2.2	22	4.1	14
PYK2	-1	0	4.6	9
RET	2.1	3.9	4	16
ROCK1	0.5	3.1	0	0.7
ROCK2	-1	0.8	1.4	1.5
RON	3	-1	-2	12
ROS	1.8	6.8	3.8	3.7
RSK1	0.8	1.8	0.6	8
RSK2	0.8	2.2	1.2	5.4
RSK3	0.6	1.1	1.5	5.6
RSK4	0.1	1	0.8	3.8
SGK1	1.1	-2	1.1	0.8
SGK2	2.7	1.3	2.3	0.2
SGK3	-2	-1	1.6	2.3
SIK	9.4	57	63	96
SPHK1	-4		-1	-11
SPHK2	-2	4.4	-1	-3
SRC	5.1	40	8	33
SRMS	0.8	17	-1	8.9
SRPK1	3.2	2.7	-1	-2
SYK	-0	-2	-1	-12
TBK1	1.5	3.7	4.5	16
TEC	0.3	14	1.8	5.5
TIE2	1	3	2	16
TNK1	0.4	10	18	63
TNK2	3.6	31	2	10
TRKA	1.5	2.8	1.9	7.2
TRKB	1.8	3.9	0.8	3
TRKC	2.7	2.4	1.6	3.9
TSSK1	-1	-2	3.5	5.2
TSSK2	-1	-3	0	0.5
TTK	-4	-1	4.5	7.6
TXK	2.8	36	4.1	16
TYK2	-0	1.8	-1	-2
TYRO3	1.8	19	13	58
YES	4.9	34	12	50
ZAP70	-2	1	4.7	1
